# Can we trust the experiment? Anisotropic dis­placement parameters in 1-(halometh­yl)-3-nitro­benzene (halogen = Cl or Br)

**DOI:** 10.1107/S2053229620006221

**Published:** 2020-05-20

**Authors:** Damian Mroz, Ruimin Wang, Ulli Englert, Richard Dronskowski

**Affiliations:** a Institute of Inorganic Chemistry, RWTH Aachen University, Landoltweg 1, 52056 Aachen, Germany; b Institute of Molecular Science, Shanxi University, 030006 Taiyuan, Shanxi, People’s Republic of China; c Jülich-Aachen Research Alliance (JARA-HPC), Forschungszentrum Jülich, 52056 Aachem, Germany; d Hoffmann Institute of Advanced Materials, Shenzhen, Liuxian 7098, People’s Republic of China

**Keywords:** density functional theory, DFT, anisotropic displacement parameters, ADP, phonon calculations, mol­ecular crystal, crystal structure, synchrotron

## Abstract

1-(Chloro­meth­yl)-3-nitro­benzene, C_7_H_6_NClO_2_, and 1-(bromo­meth­yl)-3-nitro­benzene, C_7_H_6_NBrO_2_, were chosen as test compounds for benchmarking anisotropic displacement parameters (ADPs) calculated from first principles in the harmonic approximation. Crystals of these compounds are isomorphous, and theory predicted similar ADPs for both. In-house diffraction experiments with Mo *K*α radiation were in apparent contradiction to this theoretical result, with experimentally observed ADPs significantly larger for the bromo derivative. In contrast, the experimental and theoretical ADPs for the lighter congener matched reasonably well. As all usual quality indicators for both sets of experimental data were satisfactory, complementary diffraction experiments were performed at a synchrotron beamline with shorter wavelength. Refinements based on these intensity data gave very similar ADPs for both compounds and were thus in agreement with the earlier in-house results for the chloro derivative and the predictions of theory. We speculate that strong absorption by the heavy halogen may be the reason for the observed discrepancy.

## Abstract 2

Anisotropic displacement parameters for the isomorphous compounds 1-(halometh­yl)-3-nitro­benzene (halo = chloro and bromo) were calculated from first principles and determined by X-ray diffraction experiments. Unexpectedly, the experiment for the bromo compound proved more challenging than theory.

## Introduction   

Careful diffraction experiments on crystals of reasonable quality provide reliable intensity data from which atomic positions and anisotropic displacement parameters (ADPs) can be derived almost routinely. The alternative route towards ADPs, namely, their calculation from first principles, has made good progress (George *et al.*, 2015*a* ▸,*b* ▸, 2016 ▸, 2017 ▸; Deringer *et al.*, 2014 ▸, 2016 ▸, 2017 ▸; Baima *et al.*, 2016 ▸; Lane *et al.*, 2012 ▸; Madsen *et al.*, 2013 ▸; Pozzi *et al.*, 2013 ▸; Dittrich *et al.*, 2012 ▸).

**Figure 1:**
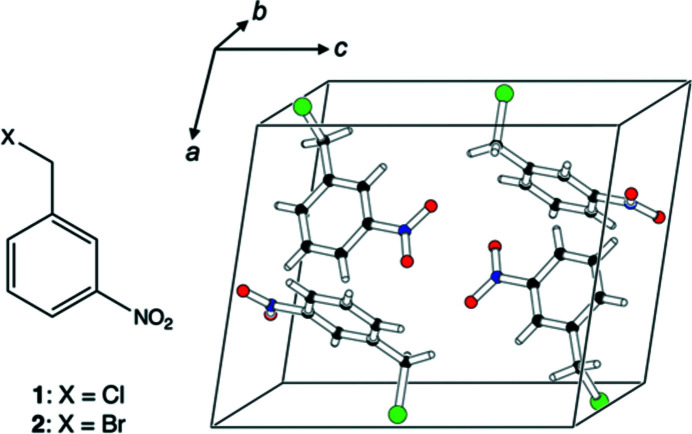
Chemical diagram (left) for 1-(chloro­meth­yl)-3-nitro­benzene (**1**) and 1-(bromo­meth­yl)-3-nitro­benzene (**2**), and the unit cell (right) of **1** at 100 K based on an in-house single-crystal X-ray diffraction experiment (data set **1a**).

This progress has been benchmarked by comparison with the results from single-crystal X-ray or neutron diffraction. In this context, a ‘heavy atom problem’ with ADPs from theory was suspected (Deringer *et al.*, 2016 ▸) but not conclusively proven. We therefore decided to calculate the ADPs in two iso­morphous (Authier & Chapuis, 2014 ▸; IUCr Online Dic­tionary of Crystallography, 2017 ▸) organic crystals and com­pare the results from theory to their experimental counterparts. The nitro­aromatic compounds 1-(chloro­meth­yl)-3-nitro­ben­zene, **1**, and 1-(bromo­meth­yl)-3-nitro­benzene, **2** (Fig. 1 ▸), were identified as suitable test candidates: they share the same crystal chemistry but differ significantly with respect to the mass and electron count of the heavy atom involved, *i.e.* Cl *versus* Br.

The crystal structures of both compounds have been reported previously: a single-crystal diffraction experiment at standard resolution and room temperature was conducted on **1** [Cambridge Structural Database (CSD; Groom *et al.*, 2016 ▸) refcode PUJSUJ (Abbasi *et al.*, 2010 ▸)]. More relevant in the context of this work is the previous report on **2** (CSD refcode INEFIS; Maris, 2016 ▸) because it was based on diffraction data collected at 100 K, the same temperature as in our case; we will come back to this CSD communication in more detail below.

## Experimental   

Compounds **1** and **2** were obtained from Sigma–Aldrich and recrystallized from methanol by slow evaporation at room tem­perature. The elevated vapour pressure of these com­pounds does not permit their storage for periods longer than a few weeks. An Oxford Cryostream device was used to maintain a constant data-collection temperature of 100 K.

Synchrotron data were collected at the DESY Hamburg, beamline P24 for Chemical Crystallography at PETRA-III on the κ diffractometer (station EH1) at a photon energy of 20 keV (λ = 0.61992 Å). A Dectris CdTe 1M area detector was used and the exposure time per frame was 5 s. Data were processed with *XDS* (Kabsch *et al.*, 2010 ▸) and corrected for absorption with *SADABS* (Bruker, 2015 ▸).

H atoms were introduced in calculated positions and treated as riding, with C—H distances of 0.95 (aromatic) or 0.99 Å (methyl­ene) and with *U*_iso_(H) = 1.2*U*_eq_(C). Crystal data, data collection parameters and key quality indicators have been compiled in Table 1 ▸.

Electronic-structure calculations based on density-functional theory (DFT) were performed using the Vienna *ab initio* simulation package (Version 5.4.4) (Kresse & Hafner, 1993 ▸, 1994 ▸; Kresse & Furthmüller, 1996*a* ▸,*b* ▸). The PBE functional (Perdew *et al.*, 1996 ▸), in conjunction with the projector-augmented wave method (Kresse & Joubert, 1999 ▸; Blöchl, 1994 ▸), were utilized. Additionally, the D3 dispersion correction of Grimme and co-workers in combination with Becke–Johnson damping was used to account for van der Waals inter­actions (Grimme *et al.*, 2010 ▸, 2011 ▸). The kinetic energy cutoff of the plane wave expansion was limited to 500 eV.

The structures under investigation were optimized with respect to the energy, using a convergence criterion of 10^−6^ eV with regard to the structural optimization and 10^−8^ eV for the electronic steps. After checking the *k*-point convergence in the calculations, supercells were created based on the optimized structures with *Phonopy* (Togo *et al.*, 2008 ▸; Togo & Tanaka, 2015 ▸). All supercells had a length of at least about 15 Å in each direction. The subsequent phonon calculations were performed with 27 × 62 × 22 *q*-points for both structures, concerning the phononic DOS (density of phonon states, DPS) and thermal displacements, as implemented in *Phonopy*, while using a frequency cutoff of 0.1 THz. A finite displacement (Parlinski *et al.*, 1997 ▸) of 0.01 Å was used for the calculations, as mentioned above. However, it should be noted that the supercell calculations were only performed at the Γ point. The conversion of the crystallographic coordinates to Cartesian coordinates (Grosse-Kunstleve & Adams, 2002 ▸) was per­formed by a custom-made program, namely, the *Mol­ecular Toolbox* (George, 2016 ▸), written in MATLAB (MATLAB, 2016 ▸). Moreover, this program was used to calculate the root-mean-square of the Cartesian deviations (RMS) (George *et al.*, 2014 ▸).

The quasiharmonic approximation (Stoffel *et al.*, 2010 ▸) was also used by optimizing the initial structure for various compression and expansion factors of the unit-cell volume. This procedure was carried out in steps of 0.01 in the range from 0.96 to 1.04. The subsequent phonon calculations were performed as described above. After calculating the thermal properties and energies, the Vinet equation of state (Vinet *et al.*, 1987 ▸), as implemented in *Phonopy*, was used to predict the thermal expansion of the system at 100 K. The following steps were performed as described above for the harmonic case, but with a unit cell relaxed under the estimated thermal expansion.

## Results and discussion   

Our initial diffraction experiments were conducted with in-house equipment at 100 K. Mo Kα radiation from a microfocus tube was used, and data collections extended to a resolution of 0.62 Å (λ_max_ = 35°). We will refer to these data sets as **1a** and **2a**. A first comparison between the experimental and energy-minimized crystal structures in terms of lattice parameters and overall residuals of mean Cartesian displacements (RMS) is provided in Table 2 ▸ and documents a good match.

Lattice parameters of the minimum energy structures match those observed experimentally equally well for **1** and **2**, but a different picture is obtained when displacement parameters are considered. At low temperatures, such as 100 or 150 K, theoretical ADPs from first principles based on the harmonic approximation can be expected to match experiments reasonably well (George *et al.*, 2015*a* ▸,*b* ▸; Deringer *et al.*, 2016 ▸; Mroz *et al.*, 2019 ▸).

**Figure 2:**
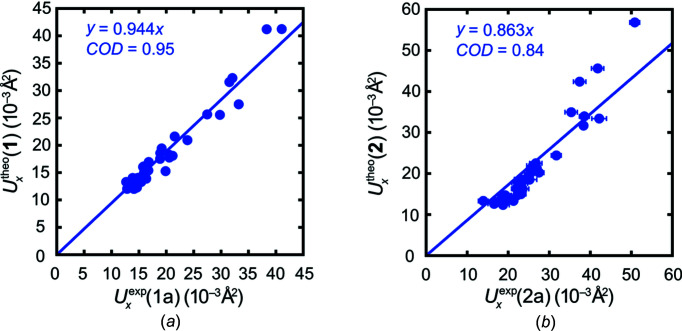
Scatter plots of the theoretical and experimental main-axis components *U_x_* (*x* = 1, 2 or 3) with linear fits and coefficients of determination (CODs) for 100 K in the harmonic approximation. The superscript notation ‘exp’ denotes experimental values and ‘theo’ stands for theoretical values. (*a*) Plot for compound **1**, data set **1a**. (*b*) Plot for compound **2**, data set **2a**.

**Figure 3:**
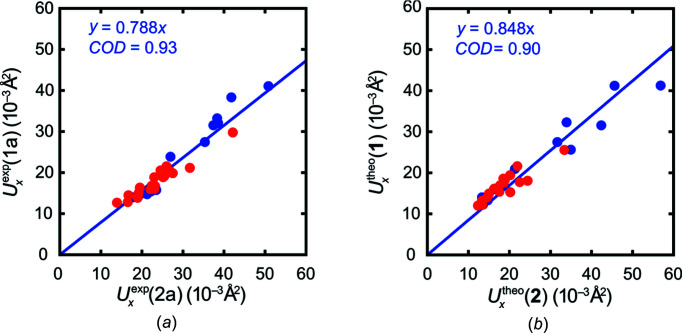
(*a*) Scatter plot of the experimental main-axis components derived from data set **1a** *versus* the experimental main-axis components derived from data set **2a**. (*b*) Analogous to the scatter plot in part (*a*), but now correlating theoretical results for **1** with those for **2**. The main axes components of the C atoms are highlighted in red. All other atoms are portrayed in blue.

Fig. 2 ▸ shows that this is only true for the home-lab data associated with chloro derivative **1** because the slope (0.944) is close to unity; for heavy-atom structure **2**, the apparent underestimation by theory *versus* data set **2a** (slope = 0.863) is more pronounced than expected. This trend can alternatively be visualized when the experimental ADPs for both isomorphous compounds are correlated with each other (Fig. 3 ▸). The lower slope of 0.788 in this figure indicates that the experimental ADPs derived from **1a**, designated as *U_x_*(**1a**), stay smaller than those obtained from **2a**, given as *U_x_*(**2a**), throughout. If we trust in the home-lab data collected on the diffractometer and the same (low) temperature of 100 K, the ADPs for **2** are significantly larger than for the lighter congener **1**. Despite their very close structural relationship, these compounds represent two solids with different composition: one may not reasonably expect ‘the same’ displacement for both! Experimental differences as large as those indicated by Figs. 2 ▸ and 3 ▸(*a*), however, must necessarily raise suspicion. Inter­estingly enough, theory predicts (Fig. 3 ▸ *b*) more similar displacements (slope = 0.848) for both isomorphous com­pounds, especially for the less peripheral C atoms, depicted as red data points, which lie close to the diagonal of this subfigure. It should be noted that the threefold standard uncertainties of the experimental values are too small to be visible.

In addition to this semiqu­anti­tative tool of comparison, the quasiharmonic approximation was tested for compound **2**; the results are shown in Fig. S1 (see supporting information). Here, one finds the expected result that the quasiharmonic approximation improves on the amplitude of the ADPs by incorporating temperature effects, thereby leading to larger values. However, in this case, the approach results in a clear overestimation (slope = 1.185), as also frequently seen (George *et al.*, 2017 ▸).

In view of the marked discrepancy between the ADPs derived from data sets **1a** and **2a**, the question arises whether our experimental data are sufficiently reliable to benchmark our theoretical results and diagnose a potential ‘heavy atom’ problem. They might also be affected by systematic errors, in particular when the high absorption of the atom type Br in **2** for Mo *K*α radiation (data set **2a**) is taken into account.

**Figure 4:**
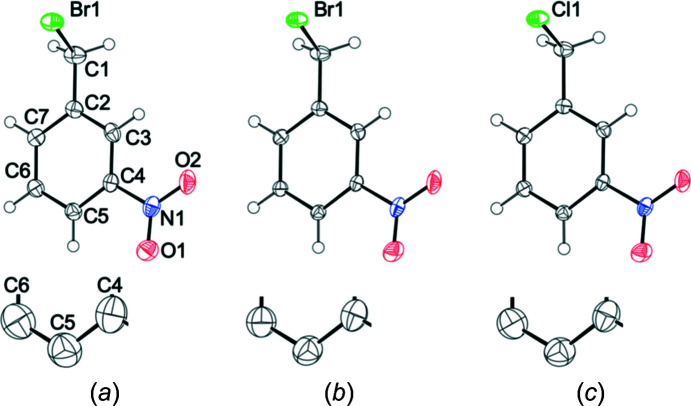
Displacement ellipsoid plots (50% probability for the complete mol­ecules and 90% probability for the magnification at the bottom showing atoms C4, C5 and C6) based on experimental diffraction data. (*a*) ADPs for **2**, based on data set **2a**, Mo *K*α radiation; (*b*) ADPs for **2**, based on data set **2b**, synchrotron radiation (λ = 0.61992 Å); (*c*) ADPs for **1**, based on data set **1a**, Mo *K*α radiation.

Two potentially relevant aspects of absorption may be addressed at the same time when home-lab Mo *K*α radiation is replaced by a shorter wavelength at a synchrotron; the shorter wavelength will lead to a lower linear absorption coefficient for **2**, and the high flux of the synchrotron will allow the use of significantly smaller crystals. Our experiments were conducted at beamline P24 of the DESY; we will refer to the resulting intensity data as **1b** and **2b**. The synchrotron facility eliminated another possible systematic error with the experimental data: modern radiation sources, such as microfocus and metal jet sources, typically produce beams of a small diameter at the sample position, whereas P24 optics ensure that even large crystals of 0.2 mm are completely illuminated. We will come back to this aspect below. Fig. 4 ▸ compiles displacement ellipsoid plots for **2** and **1** based on experimental diffraction data. Clearly, the too-large ADPs of **2** as given by the laboratory data **2a** using Mo *K*α radiation (Fig. 4*a* ▸) become significantly smaller using synchrotron radiation (**2b**; Fig. 4*b* ▸), and then they resemble those of **1** based on data set **1a** obtained with Mo *K*α radiation (Fig. 4*c* ▸).

**Figure 5:**
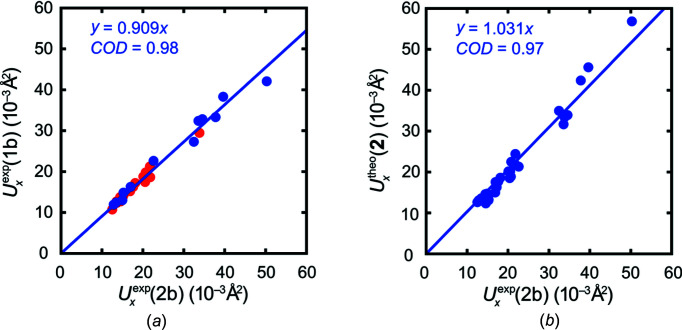
(*a*) Scatter plot of experimental main-axis components derived from data set **1b** *versus* the experimental main-axis components derived from data set **2b**. The main axes components of the C atoms are highlighted in red. All other atoms are portrayed in blue. (*b*) Correlation of the theoretical results for **2** with the synchrotron data **2b**.

In more general terms, Fig. 5 ▸ evidences that ADPs based on intensity data collected at the synchrotron compare much more favourably to theory in terms of absolute numbers (mirrored from the slopes). Additionally, the correlation between the two experimental data sets stemming from synchrotron measurements is also more satisfying.

As an additional test, the diffraction experiment on **1** was repeated at the synchrotron; both home lab (**1a**) and synchrotron data (**1b**) are almost superimposable. The corresponding correlation is shown in the supporting information (Fig. S2). Moreover, the supporting information contain more details of further theoretical results.

**Figure 6:**
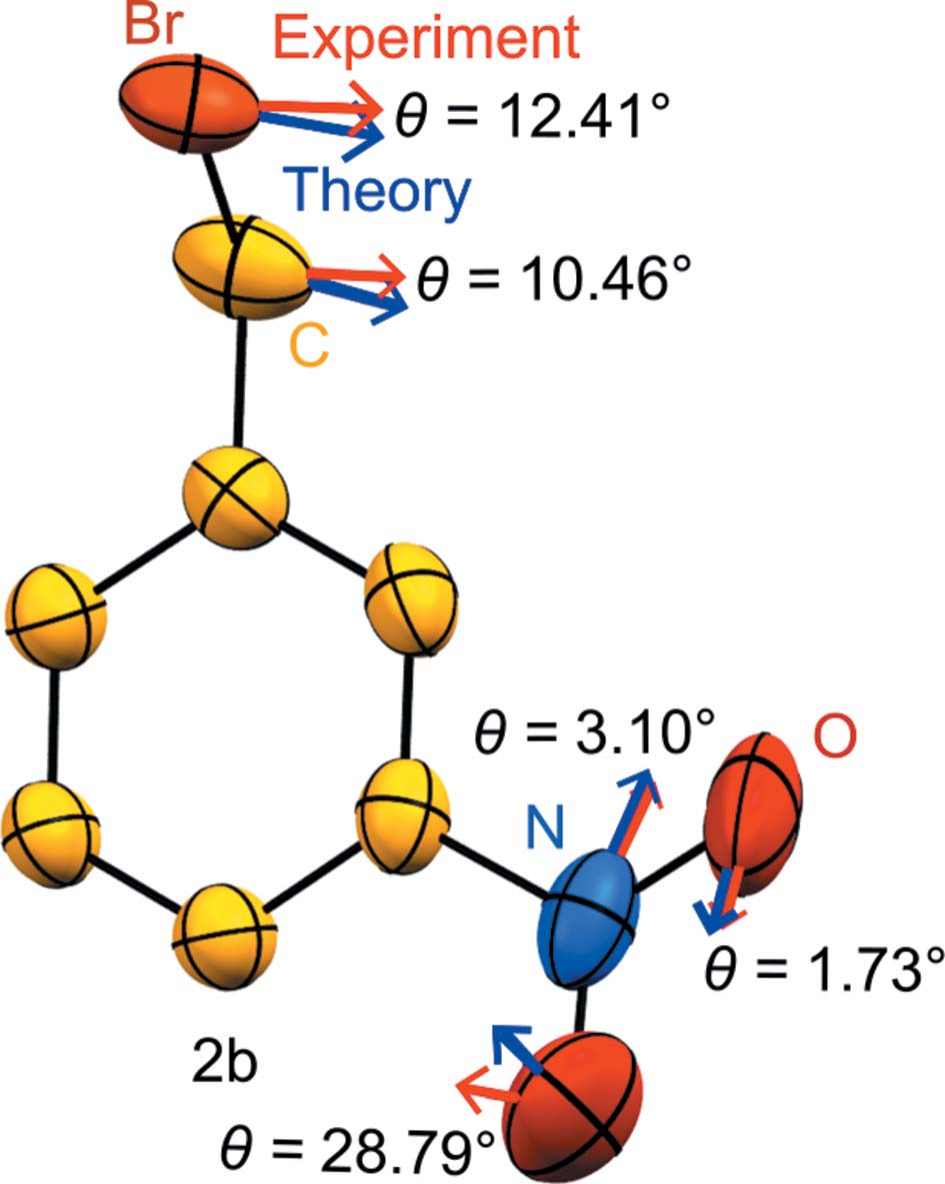
Comparison between sufficiently anisotropic displacement ellipsoids (*U*_max_/*U*_min_ > 1.8) and the resulting angles between the largest theoretical (blue) and experimental (red) main-axes components for data set **2b** at 100 K. ADPs are derived from intensity data collected at the synchrotron. The structure is drawn in the theoretically predicted coordinate system.

It is important to note that the results of both diffraction experiments on **2**, at the usual Mo *K*α home source and at the synchrotron, result in ADPs which comply with Hirshfeld’s rigid bond test (Hirshfeld, 1976 ▸), a well-established requirement for mol­ecular crystals. Even better, the ADPs derived from both data collections *agree* with respect to the essential message about the main directions of mol­ecular motion, whereas their disagreement largely corresponds to the amplitudes. We have recently suggested (Mroz *et al.*, 2019 ▸) that the directionality of sufficiently prolate ADPs provides a simple way to visually compare the main modes of thermal movement suggested by theory and experiment. The corresponding synoptic picture for the alternative diffraction data on **2** is provided in Fig. 6 ▸. The analogous analysis of ADP directionality for **1** has been compiled in the supporting information.

Fig. 6 ▸ shows that the agreement between the theoretical ADPs for **2** and the experimental ones derived from synchrotron experiments (data set **2b**) is satisfying. The only qualitative exception occurs for one O atom where the resulting angle is slightly larger than usual but still in a reasonable range, and such a deviation is not too surprising. The corresponding picture for the data set based on Mo *K*α radiation is shown in the supporting information. They essentially differ with respect to size, with a ratio *U*_eq_(Mo *K*α):*U*_eq_(sync) = 1.22 (2), whereas the correspondence of the directions is qualitatively the same. The results of an earlier diffraction experiment on **2** are available as a private communication (CSD refcode INEFIS; Maris, 2016 ▸). This diffraction experiment was performed with Ga *K*α radiation from a metal jet source at 100 K, *i.e.* at the same temperature as our data collections. Both unit-cell volume and geometry confirm this published data-collection temperature. Similar ADPs might therefore be expected, but the displacement parameters from INEFIS are about twice as large as ours. We are not in a position to give a reliable inter­pretation of the apparent trend *U*_eq_(sync) < *U*_eq_(Mo *K*α) << *U*_eq_(Ga *K*α), but it is tempting to speculate about possible reasons. When the linear absorption coefficients (μ) for the different wavelengths and the sample sizes (*r*) in all three experiments are taken into account, we find μ*r*(sync) < μ*r*(Mo *K*α) < μ*r*(Ga *K*α). Moreover, one might expect different illumination for the three samples, with the largest beam diameter at the present setup of synchrotron beamline P24 and the smallest one for the metal jet. Hence, the very large ADPs seen in INEFIS might be an artefact going back to strong absorption and insufficient illumination, but this hypothesis needs independent experimental verification. Multi-scan absorption corrections, such as those employed here, have become the *de facto* standard for diffraction data collected with area detectors. As these techniques rely on the comparison between symmetry-equivalent intensities, they necessarily require an elevated redundancy; this journal suggests at least a fourfold multiplicity of observations for multi-scan corrections. As symmetry-equivalent reflections necessarily share the same diffraction angle, a second quasi-spherical correction has to account for the 2θ dependence of absorption. Both corrections together, *i.e.* for very high redundancies and with a perfectly chosen quasi-radius for the spherical correction, should ideally correspond to the classical analytical absorption correction (de Meulenaer & Tompa, 1965 ▸) based on indexed crystal faces. The latter approach only corrects for absorption and requires complete illumination of the crystal; additional corrections, *e.g.* for crystal decay, may be performed independently. In contrast, multi-scan corrections can to a certain extent even handle variable illumination or crystal decay *via* a (restrained) incident beam scale factor (Krause *et al.*, 2015 ▸). If one wants to establish to what extent either absorption or variable illumination are responsible for apparent ADP problems, diffraction data on the same crystal should be collected as a function of beam size and wavelength, and multi-scan corrections should be tested as a function of multiplicity of observations. If the aim are benchmark ADPs, absorption and incomplete illumination should be avoided.

## Conclusions   

We set out to benchmark ADPs based on dispersion-corrected DFT calculations on the harmonic approximation, and it turned out that our in-house experiment, despite elevated redundancy and resolution, was not really able to do so. An alternative experiment at a synchrotron beamline at the same temperature but on a smaller crystal and with a short wavelength gave results in better agreement with theory. We do not dwell on compiling all possible sources of error but rather draw three optimistic conclusions: (i) the quality of theoretically calculated ADPs may challenge that of standard experiments, (ii) the directionality of the ADPs based on the intensity data of our in-house diffractometer match that obtained at the synchrotron beamline even if the amplitudes do not agree and (iii) for the (many!) crystal structures with minor absorption effects only, ADPs from good in-house data match those obtained at the synchrotron beamline; compound **1**, with its unexceptional absorption properties, provides a good example for that statement. In our future work, we will attempt to gain insight into the various sources of experimental error. The calculation of absorption-affected data by analytical methods, followed by their treatment with a multi-scan correction program might be a suitable approach.

## Supplementary Material

Crystal structure: contains datablock(s) 1a, 1b, 2a, 2b, global. DOI: 10.1107/S2053229620006221/sk3749sup1.cif

Structure factors: contains datablock(s) 1a. DOI: 10.1107/S2053229620006221/sk37491asup2.hkl

Structure factors: contains datablock(s) 1b. DOI: 10.1107/S2053229620006221/sk37491bsup3.hkl

Structure factors: contains datablock(s) 2a. DOI: 10.1107/S2053229620006221/sk37492asup4.hkl

Structure factors: contains datablock(s) 2b. DOI: 10.1107/S2053229620006221/sk37492bsup5.hkl

Extra figures and tables. DOI: 10.1107/S2053229620006221/sk3749sup6.pdf

CCDC references: 2002492, 2002491, 2002490, 2002489

## Figures and Tables

**Table 1:** Experimental details For all structures: monoclinic, *P*2_1_/*c*, *Z* = 4. Experiments were carried out at 100 K. H-atom parameters were constrained.

	**1**		**2**	
	**1a**	**1b**	**2a**	**2b**
Crystal data				
Chemical formula	C_7_H_6_ClNO_2_		C_7_H_6_BrNO_2_	
*M*_r_	171.58		216.04	
*a*, *b*, *c* (Å)	11.7867 (11), 4.4744 (4), 15.0453 (14)	11.785 (4), 4.4690 (9), 15.004 (4)	12.1412 (5), 4.4763 (2), 15.0876 (6)	12.152 (9), 4.470 (3), 15.070 (11)
β (°)	112.464 (7)	112.537 (6)	112.626 (3)	112.56 (2)
*V* (Å^3^)	733.26 (12)	729.9 (3)	756.87 (6)	756.0 (9)
Radiation type	Mo *K*α	Synchrotron, λ = 0.61992 Å	Mo *K*α	Synchrotron, λ = 0.61992 Å
μ (mm^−1^)	0.46	0.32	5.37	3.76
Crystal size (mm)	0.28 × 0.17 × 0.04	0.12 × 0.10 × 0.04	0.23 × 0.22 × 0.04	0.10 × 0.06 × 0.04

Data collection				
Diffractometer	Stoe STADIVARI with a DECTRIS Pilatus 200K detector	Kappa diffractometer (EH1) with Dectris CdTe area detector	Stoe STADIVARI with a DECTRIS Pilatus 200K detector	Kappa diffractometer (EH1) with Dectris CdTe area detector
Absorption correction	Multi-scan [*LANA* (Blessing, 1995 ▸; Koziskova *et al.*, 2016 ▸) in *X-AREA* (Stoe & Cie, 2017 ▸)]	Multi-scan (*SADABS*; Bruker, 2015 ▸)	Multi-scan [*LANA* (Blessing, 1995 ▸; Koziskova *et al.*, 2016 ▸) in *X-AREA* (Stoe & Cie, 2017 ▸)]	Multi-scan (*SADABS*; Bruker, 2015 ▸)
*T*_min_, *T*_max_	0.545, 1.000	0.728, 0.863	0.302, 1.000	0.604, 0.747
No. of measured, independent and observed [*I* > 2σ(*I*)] reflections	34338, 3231, 2604	19608, 3194, 2947	37127, 3332, 2062	18687, 3233, 3018
*R*_int_	0.035	0.110	0.165	0.056
(sin θ/λ)_max_ (Å^−1^)	0.807	0.807	0.807	0.806

Refinement				
*R*[*F* ^2^ > 2σ(*F* ^2^)], *wR*(*F* ^2^), *S*	0.030, 0.085, 1.06	0.043, 0.122, 1.07	0.037, 0.075, 1.09	0.033, 0.090, 1.10
No. of reflections	3231	3194	3332	3233
No. of parameters	100	101	101	101
Δρ_max_, Δρ_min_ (e Å^−3^)	0.52, −0.23	0.63, −0.48	0.93, −0.60	0.91, −1.29

Computer programs: *PILATUS*, *RECIPE*, *INTEGRATE* and *LANA* in *X-AREA* (Stoe & Cie, 2017 ▸), *XDS* (Kabsch *et al.*, 2010 ▸), *SHELXS97* (Sheldrick, 2008 ▸) and *SHELXL2018* (Sheldrick, 2015 ▸).

**Table 2:** Experimental (exp) and theoretically (theo) predicted lattice parameters, monoclinic angle, volume of the unit cell and root-mean-square (RMS) values of Cartesian deviations

	**1**		**2**	
	Exp*	Theo	Exp*	Theo
*a* (Å)	11.7867 (11)	11.9202	12.152 (9)	12.2703
*b* (Å)	4.4744 (4)	4.3898	4.470 (3)	4.3909
*c* (Å)	15.0453 (14)	15.0807	15.070 (11)	15.1254
β (°)	112.464 (7)	112.584	112.56 (2)	112.621
RMS	0.0920		0.0943	
*V* (Å^3^)	733.26 (12)	728.63	756.0 (9)	752.23

(*) Experimental values for compound **1** are based on in-house intensities (**1a**) and those for compound **2** stem from synchrotron intensities (**2b**).
