# Supplementary material for: Can we trust the experiment? Anisotropic dis­placement parameters in 1-(halometh­yl)-3-nitro­benzene (halogen = Cl or Br)
Source: Acta Crystallogr C Struct Chem. 2020 May 20;76(Pt 6):591–7. doi: 10.1107/S2053229620006221 (PMC7273188; doi:10.1107/S2053229620006221)
Supplement: Supplementary file 6 [file c-76-00591-sup6.pdf]

Supporting information

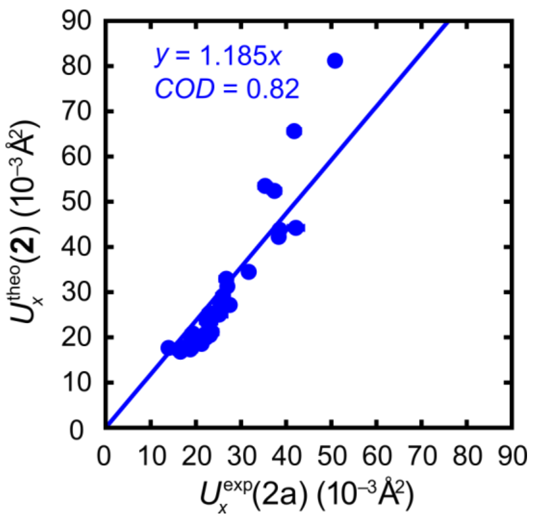

Figure S1 Scatter plot of the main axes components in the quasiharmonic approximation for data set 2a at 100 K.

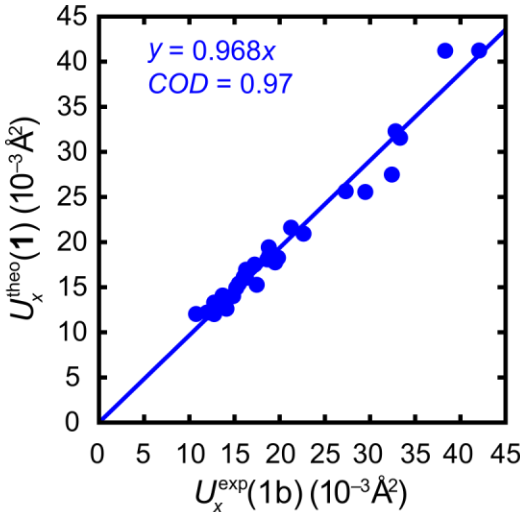

Figure S2 Scatter plots of theoretical and experimental main-axis components with linear fits and coefficients of determination (CODs) for 100 K in the harmonic approximation for data set 1b. The data stem from synchrotron measurements.

IMPORTANT: this document contains embedded data - to preserve data integrity, please ensure where possible that the IUCr Word tools (available from http://journals.iucr.org/services/docxtemplate/) are installed when editing this document.

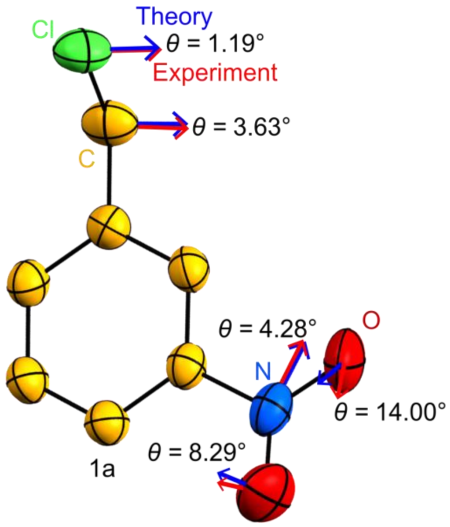

Figure S3 Angles between the largest main axes components of theoretical and experimental displacement ellipsoids for compound **1** at 100 K. The experimental data stem from X-ray intensities, data set 1a.

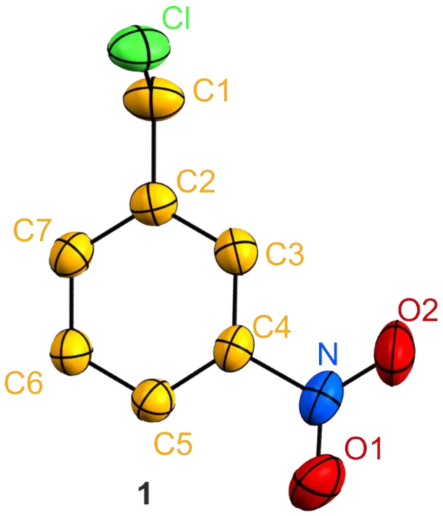

Figure S4 Thermal ellipsoids in the harmonic approximation of **1** at 100 K.

Table S1 ADPs for **1** in the harmonic approximation at 100 K. The nomenclature follows Fig S4.

Cl1	0.014110	0.024480	0.022765	0.003340	0.005877	0.000944
O1	0.034097	0.036230	0.017092	0.008048	0.011325	0.005294
O2	0.032928	0.036960	0.017951	-0.007905	0.012110	0.002249
N1	0.019550	0.022110	0.013565	-0.001947	0.007764	-0.004627
C1	0.018301	0.015790	0.022125	0.003831	0.005737	0.000892
C2	0.014540	0.013890	0.015240	0.000181	0.005918	-0.000641
C3	0.015918	0.016350	0.015738	-0.001991	0.007383	0.000120
C4	0.015116	0.016370	0.013377	-0.001059	0.006651	-0.001755
C5	0.015715	0.018690	0.015258	0.001029	0.006783	0.001495
C6	0.016791	0.020370	0.016076	-0.000082	0.008649	0.002434
C7	0.016648	0.018250	0.013916	0.000250	0.007439	-0.000070

Table S2 Angles between the largest main axes components of theoretical and experimental thermal ellipsoids for **1** at 100 K. The experimental data are based on synchrotron measurements. The nomenclature follows Fig S4.

Atom	θ (°)
Cl	2.25
C1	2.26
N	0.86
O1	5.89
O2	9.37

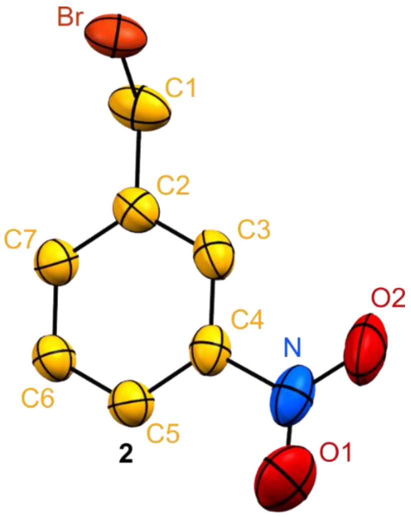

Figure S5 As in Fig. S4, but for compound **2**.

Table S3 As in Tab. S1, but for compound **2**. The nomenclature follows Fig. S5.

Br1	0.013740	0.024080	0.027992	0.004671	0.007475	0.001499
O1	0.044306	0.041430	0.019940	0.007732	0.015629	0.002054
O2	0.034353	0.049890	0.022878	-0.015346	0.014630	-0.002743
N1	0.022883	0.027860	0.016854	-0.005077	0.010751	-0.008902
C1	0.018506	0.015700	0.030685	0.004472	0.007823	0.000749
C2	0.014837	0.014160	0.020151	-0.000469	0.007447	-0.000947
C3	0.017066	0.018140	0.020854	-0.004601	0.009729	-0.001196
C4	0.016776	0.018740	0.016331	-0.002885	0.008712	-0.003671
C5	0.016942	0.019810	0.016865	0.000809	0.007883	0.000673
C6	0.016950	0.020800	0.017500	-0.000004	0.009284	0.002306
C7	0.016474	0.018750	0.016433	0.000551	0.008142	-0.000138

Table S4 As in Tab. S3, but in the quasiharmonic approximation at 100 K.

Br1	0.019069	0.034050	0.038082	0.004900	0.010189	0.001677
O1	0.057311	0.056960	0.025654	0.008473	0.018314	-0.005385
O2	0.046686	0.070780	0.030605	-0.022131	0.020802	-0.009665
N1	0.032454	0.041860	0.022410	-0.007611	0.014518	-0.015440
C1	0.025042	0.021640	0.041131	0.004789	0.010754	0.000842
C2	0.020225	0.019320	0.026920	-0.001401	0.010438	-0.001853
C3	0.023139	0.025160	0.027965	-0.007320	0.013589	-0.003338
C4	0.023064	0.026830	0.021695	-0.004484	0.011674	-0.006958
C5	0.022794	0.027030	0.022822	0.000849	0.009913	-0.000618
C6	0.022739	0.028120	0.023845	-0.000598	0.012197	0.002548
C7	0.022115	0.025300	0.021804	0.000029	0.011053	-0.000633

Table S5 As in Tab. S2, but for compound **2**. The experimental data stem from X-ray measurements. The nomenclature follows Fig. S4.

Atom	θ (°)
Br	3.85
C1	10.61
N	1.59
O1	4.82
O2	6.73

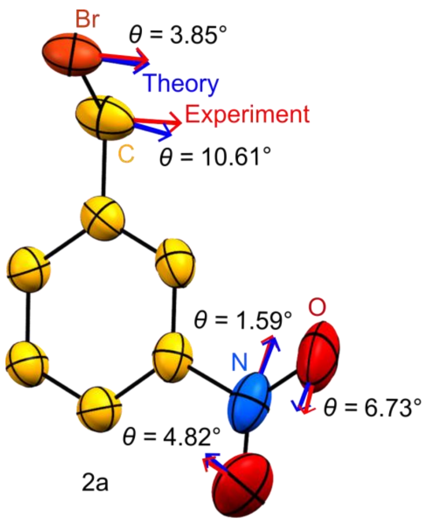

Figure S6 As in Fig S3., but for data set 2a.
